# Identifying and classifying goals for scientific knowledge

**DOI:** 10.1093/bioadv/vbab012

**Published:** 2021-07-28

**Authors:** Mayla R Boguslav, Nourah M Salem, Elizabeth K White, Sonia M Leach, Lawrence E Hunter

**Affiliations:** 1 Computational Bioscience Program, University of Colorado Anschutz Medical Campus, Aurora, CO 80045, USA; 2 Health Informatics Program, College of Health Solutions at Arizona State University, Phoenix, AZ 85004, USA; 3 Center for Genes, Environment and Health, National Jewish Health, Denver, CO 80206, USA

## Abstract

**Motivation:**

Science progresses by posing good questions, yet work in biomedical text mining has not focused on them much. We propose a novel idea for biomedical natural language processing: identifying and characterizing the *questions* stated in the biomedical literature. Formally, the task is to identify and characterize *statements of ignorance*, statements where scientific knowledge is missing or incomplete. The creation of such technology could have many significant impacts, from the training of PhD students to ranking publications and prioritizing funding based on particular questions of interest. The work presented here is intended as the first step towards these goals.

**Results:**

We present a novel ignorance taxonomy driven by the role statements of ignorance play in research, identifying specific goals for future scientific knowledge. Using this taxonomy and reliable annotation guidelines (inter-annotator agreement above 80%), we created a gold standard ignorance corpus of 60 full-text documents from the prenatal nutrition literature with over 10 000 annotations and used it to train classifiers that achieved over 0.80 F1 scores.

**Availability and implementation:**

Corpus and source code freely available for download at https://github.com/UCDenver-ccp/Ignorance-Question-Work. The source code is implemented in Python.

## 1 Introduction

Posing good questions is as fundamental to scientific progress as analysing experimental results (Firestein, 2012; Kuhn, 2012; O’leary, 2004). Until now, biomedical text mining has focused on extracting information from the literature (Cambria and White, 2014) either ignoring (Farkas *et al.*, 2010; Ganter and Strube, 2009; Medlock and Briscoe, 2007; Vincze *et al.*, 2008) or de-emphasizing (Kilicoglu *et al.*, 2017; Kilicoglu and Bergler, 2008; Light *et al.*, 2004; Zerva *et al.*, 2017) questions and other statements regarding uncertainty or missing knowledge. However, the scientific literature is full of statements about knowledge that does not exist yet, including goals for desired knowledge, statements about uncertainties in interpretation of results, discussions of controversies and many others; collectively, we term these *statements of ignorance*, borrowing the term from Firestein (2012). Philosophers of science have attended to statements of ignorance as a driving force in the selection of research topics and approaches (Bromberger, 1992; Firestein, 2012; Han *et al.*, 2011; Kuhn, 2012; Martin and White, 2003; Pearl and Mackenzie, 2018; Regan *et al.*, 2002; Rubin, 2006; Smithson, 2012; Walker, 1990). Furthermore, they are the subject of many passages in nearly every biomedical publication, even when not stated explicitly as a syntactic question (Firestein, 2012; Kuhn, 2012; O’leary, 2004). Our goal is to identify and parse a research article into its statements of ignorance [similar to the decomposition of complex questions in the Question Answering literature (Perez *et al.*, 2020)]. Here we describe a novel natural language processing (NLP) task: identification and characterization of statements of ignorance. We present an estimate of the extent of statements of ignorance in a sample of the full-text biomedical literature, provide a theoretically driven taxonomy of such statements, describe a manually annotated corpus of statements of ignorance and their categorization (along with novel annotation guidelines for the task), and demonstrate that automatically identifying and classifying statements of ignorance is feasible.

An automated approach to identifying and characterizing statements of ignorance has a variety of significant use cases. One is to facilitate interdisciplinary interactions: a collection of formally characterized statements of ignorance could identify questions from other disciplines that new results might bear on, particularly when considering genome-scale data such as transcriptomics (e.g. Rihm *et al.*, 2003). A systematic survey of scientific questions could be useful to a wide variety of scientists, ranging from graduate students looking for thesis projects (e.g. Tanaka, 2020) to funding agencies tracking emerging research areas (e.g. Boyack and Börner, 2003). Another potential application is longitudinal analysis, for example, tracking the evolution of research questions over time (e.g. Balili *et al.*, 2017). Furthermore, identifying questions would allow us to query existing databases for information (e.g. Faruqui and Das, 2018). However, in all the previous attempts at such applications, statements of ignorance are not the focus, and thus they lack grounding in the broader research field.

Although this task is novel, it is related to prior NLP efforts to characterize uncertainty, hedging, speculation and meta-knowledge (Bandrowski *et al.*, 2016; Bastian *et al.*, 2015; Brush *et al.*, 2016; Carter *et al.*, 2016; Chen *et al.*, 2018; Chibucos *et al.*, 2014; Farkas *et al.*, 2010; Ganter and Strube, 2009; Jean *et al.*, 2016; Kilicoglu *et al.*, 2017; Kilicoglu and Bergler, 2008; Light *et al.*, 2004; Medlock and Briscoe, 2007; Patrick and Li, 2012; Shardlow *et al.*, 2018; Vincze *et al.*, 2008; Yang *et al.*, 2012; Zerva *et al.*, 2017). It also draws from related work in the philosophy of science regarding how scientists choose problems and approaches, which requires that they characterize their goals for new knowledge (Bromberger, 1992; Firestein, 2012; Han *et al.*, 2011; Kuhn, 2012; Martin and White, 2003; Pearl and Mackenzie, 2018; Regan *et al.*, 2002; Rubin, 2006; Smithson, 2012; Walker, 1990).

While these ideas and methods are generally applicable across biomedical research, here we focus on one specific area, prenatal nutrition. This body of literature is small enough to be tractable and diverse enough to show feasibility. It is also significant to global health, since women are understudied (Holdcroft, 2007; Slawson, 2019), especially when pregnant (Mastroianni *et al.*, 2017; Meadows, 2001), due to ethical and legal considerations and complexities. We hope that even this initial pilot demonstration will be significant, as it has the potential to facilitate new interdisciplinary interactions that could advance the study of this underserved population.

### 1.1 Related work

Previous work in both NLP and more broadly provides the foundation of our approach but differs in a variety of important ways. There is no canonical name for the phenomenon we are calling *ignorance*, although prior work has used the related terms *hedging*, *uncertainty*, *speculation* and *meta-knowledge*, each of which has been defined to be somewhat different than our focus here.

NLP research has long sought to identify statements containing uncertainty, hedging and speculation. The goal of most of these efforts though is to exclude such statements from more knowledge-focused (as compared to ignorance-focused) information extraction; in contrast, we want focus on them. Note that hedging, while a related concept, does not always indicate a statement of ignorance. For instance, ‘The exact molecular function of SEPW1 protein is unknown to date’ is a statement of ignorance, but is not hedged, while ‘Thus, depending on the cellular environment, the short- and long-term effects of Tax expression can be quite different’ is hedged (Vincze *et al.*, 2008), but not a statement of ignorance.

Introducing a novel NLP task requires defining the task, determining if it is feasible for humans to perform accurately and finding ways to automate it. Researchers on hedging leveraged the linguistic definition of hedged statements [as statements that can be true or false to some extent (Lakoff, 1975)] to identify lexical cues, words or phrases that communicate uncertainty. Using these cues, they proved that hedged statements could be identified and classified in the scientific literature (Hyland, 1998; Kilicoglu and Bergler, 2008; Light *et al.*, 2004; Medlock and Briscoe, 2007), and they built corpora for hedging both in the biomedical (Rindflesch and Fiszman, 2003; Thompson *et al.*, 2011; Vincze *et al.*, 2008) and general (Ganter and Strube, 2009) domains. BioScope (Vincze *et al.*, 2008), which covers hedged and negated statements and defines their exact spans, is one of the largest such corpora in the scientific domain. With many corpora, efforts to automate the identification of hedging and scopes continued through a shared task in 2010 (Farkas *et al.*, 2010). Automation efforts included but were not limited to a Conditional Random Field (CRF) (Yang *et al.*, 2012), a probabilistic model (Jean *et al.*, 2016), and hybrid approaches (Kilicoglu *et al.*, 2017; Zerva *et al.*, 2017). We use similar methods for automating our task. Not only has work on hedges produced corpora and classification algorithms; it has also yielded formal categorizations of the phenomenon in the form of both taxonomies and ontologies. A taxonomy is a ‘hierarchy consisting of terms denoting types (or universals or classes) linked by subtype relations’ (Arp *et al.*, 2015). Relevant taxonomies for both the biomedical (Han *et al.*, 2011; Kilicoglu *et al.*, 2017; Pearl and Mackenzie, 2018; Regan *et al.*, 2002; Smithson, 2012) and general (Walker, 1990) domains exist for hedging. In terms of ontologies, at least four ontologies (Bandrowski *et al.*, 2016; Bastian *et al.*, 2015; Brush *et al.*, 2016; Chibucos *et al.*, 2014) describe the degree of evidence or confidence underpinning a statement and thus relate to our work here. However, none of these prior ontological efforts is adequate to represent the diverse family of statements of ignorance found in the scientific literature.

In contrast to the work on hedging and speculation intended to excise such statements from factual material in order to discard them, our goal is to identify statements of ignorance in scientific writing in order to analyse and use them directly in the form of knowledge goals or actionable next steps for the research. Closely related works that aim to use such statements directly focus on determining if clinical questions in patient notes are answerable or not (Patrick and Li, 2012) and attempting to link an author’s findings to statements of intended knowledge gain (although with a more limited set of knowledge goals than we pursue here; Shardlow *et al.*, 2018). Chen *et al.* (2018) though is perhaps the closest to our work here in that their overarching goal is to capture the integral role that the epistemic status of scientific propositions play in scientific change, but they do not go beyond identification. They do, however, propose scalable and adaptive methods for finding uncertainty cue words, which we build on here.

For the broader task of capturing statements of ignorance, we have taken a similar approach as previous work on hedging, adopting established lexical hedge cues and adding other lexical cues for statements about missing, incomplete, uncertain or incorrect knowledge to encompass a broader collection of ignorance phenomena. Like the BioScope corpus, our corpus defines the text spans for these lexical cues and the statements of ignorance that contain them. Like the shared task competitors, we use CRFs, probabilistic models, and hybrids of both to automatically identify statements of ignorance. We also draw on and expand existing taxonomies to construct a taxonomy of lexical cues indicating statements of ignorance. This ignorance taxonomy is broader and more diverse than the existing literature can supply. This taxonomy is further driven by knowledge goals extending previous preliminary work in the area (Chen *et al.*, 2018; Patrick and Li, 2012; Shardlow *et al.*, 2018).

## 2 Methods

**Fig. 1.**
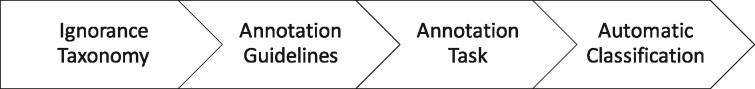
Methods flowchart. A flowchart of the methods

To achieve our goal of identifying and characterizing questions stated in the biomedical literature, we must first formalize what it means to be a statement of ignorance and the knowledge goal such a statement entails, and then demonstrate that such statements of ignorance exist and can be identified, both manually and automatically, in the literature. To do this, we (i) develop a taxonomy of types of statements of ignorance, (ii) create annotation guidelines for recognizing such statements, (iii) validate their use through an annotation task to create a manually labelled corpus, and lastly, (iv) use the corpus to benchmark classifiers to automatically recognize statements of ignorance (see Fig. 1). These resources help ground future research aimed at exploring the state of our scientific ignorance.

### 2.1 Materials

Scientific articles from our subject area of prenatal nutrition were taken from the PubMed Central Open Access (PMCOA) subset of PubMed (PMC, 2018), to ensure access to full-text articles and free sharing of data. We queried PMCOA using 54 regular expressions determined in consultation with a prenatal nutrition expert, which included keywords such as {prenatal, perinatal and antenatal} paired with keywords like {nutrition, vitamin and supplement} (the full query can be found on the GitHub page below). In total, we gathered 1 643 articles, subsets of which were used for each task below. All computation was written in Python 3, with its associated packages. In addition, the annotation task used Knowtator (Pielke-Lombardo, 2018) and Protege (Knublauch *et al.*, 2004) to annotate the full-text articles; this allows the ignorance taxonomy to be easily browsable like an ontology and helps the annotators select the correct level of specificity for each lexical cue. All code and associated materials, including the full query, taxonomy, annotation guidelines, corpus and classification models can be found here: https://github.com/UCDenver-ccp/Ignorance-Question-Work.

### 2.2 Task description

We want to produce a gold standard corpus consisting of articles with labelled sentences as *statements of ignorance* along with the *lexical cue(s)* (words or short phrases) that distinctly signify it as such mapped to a categorization of *knowledge goals (ignorance taxonomy)*. This is done through detecting spans of text either as a whole sentence or as words or short phrases. We also provide preliminary classification algorithms that aim to automate the identification of both the statements of ignorance (sentences) in an article and the specific lexical cues (words or phrases). Taking the example above, ‘<The exact molecular function of SEPW1 protein is UNKNOWN to date >’, the goal is for an annotator to identify or an algorithm to classify that this article sentence is a statement of ignorance as shown by the brackets around the sentence. From there, once the sentence is deemed a statement of ignorance, the goal is to identify or classify that UNKNOWN (shown in all caps and underlined in the example) is the lexical cue that signifies it as such. Note that one sentence can have more than one lexical cue that signifies ignorance. Thus, the ignorance taxonomy helps to distinguish between different lexical cues: the annotator and classifier also need to map the cue UNKNOWN to a specific ignorance category that captures the knowledge goal of the sentence. Following the example, the knowledge goal is to explore the exact molecular function of SEPW1 protein further to gain any insights. The taxonomy category is a *full unknown* and the word UNKNOWN would be mapped to that by the annotator and the classifier.

### 2.3 Ignorance taxonomy

*Full unknown* is only one type of ignorance where the statement indicates something is not known or there is a lack of information on a topic. To determine the different types of statements of ignorance and create the ignorance taxonomy, we first manually reviewed a subset of 736 article abstracts among the 1 643 prenatal nutrition articles from PMCOA. We conducted our task, focusing specifically on the lexical cues that signified that knowledge was missing or incomplete as in UNKNOWN in the above example. These lexical cues were then grouped together and organized into a taxonomy of lexical cues based on the knowledge goal each cue suggested. For example, the taxonomy category *full unknown* includes lexical cues such as unknown, uncertain, still unclear, could not find, etc. So each cue maps to a specific taxonomy category. Lexical cues and categories were also inspired by and added from existing work (Firestein, 2012; Han *et al.*, 2011; Kilicoglu *et al.*, 2017; Kilicoglu and Bergler, 2008; Kuhn, 2012; Pearl and Mackenzie, 2018; Smithson, 2012; Vincze *et al.*, 2008) to create an initial *ignorance taxonomy* driven by knowledge goals. The majority of lexical cues correspond to a single ignorance category, and thus imply a specific category assignment, though some cues, such as CHALLENGE, IF SO, and IMPLY appear in multiple categories and the correct category assignment then depends on the sentence context. The taxonomy is a hierarchy of both broad (a higher-level grouping of ignorance categories) and narrow categories (the ignorance category along with all lexical cues) based on the different types of knowledge goals. Lastly, the taxonomy is dynamic and iteratively updated further during the annotation task as the annotators find lexical cues beyond what was gathered during this manual review, as described below. We present the final taxonomy after all the annotations were completed.

### 2.4 Annotation guidelines

To determine if identifying statements of ignorance is reproducible and feasible, we developed annotation guidelines for annotating articles based on the manual review that not only helps annotators find statements of ignorance with lexical cues already in the ignorance taxonomy but also can help them identify new cues to add. In particular, through the manual review, we recognized the importance of lexical cues and example sentences to help guide the annotators in their decision to determine if a sentence is a statement of ignorance, the lexical cue, and the ignorance taxonomy category. The lexical cues gathered from the manual review were used to pre-process unmarked full-text articles, labelling all the cues in the text that are already pre-mapped to the ignorance taxonomy. The annotators received these pre-processed articles and for each lexical cue marked, decided if it is correct or not: determined whether the sentence containing a marked cue is indeed a statement of ignorance, if yes, they need to ensure that the ignorance category mapped to by the lexical cue is the correct one or change it, and if not then delete the pre-marked cue. Lastly, the annotators read the whole article and as our lexical cue list is not exhaustive, they can identify additional statements of ignorance and their lexical cues that are not yet part of the taxonomy and add in the new mappings.

In order to help annotators reliably make a determination if a sentence and lexical cue signify ignorance, we provided many examples of lexical cues identified in statements of ignorance from the general PMCOA. We gathered and reviewed 150 sentences to provide both positive and negative examples for each lexical cue identified. For example, ‘<however, there is CONTRADICTORY evidence from recent studies regarding the influence of IL-6 on insulin action and glucose metabolism>’ represents a statement of ignorance of an *alternative option/controversy*, where we need to resolve disagreements about the influence of IL-6. At the same time, the sentence: ‘although yin and yang are CONTRADICTORY in nature, they depend on each other for existence’ is not a statement of ignorance, although it is a negative example for the CONTRADICTORY lexical cue. Negative examples help the annotators avoid the assumption that a lexical cue necessarily entails a statement of ignorance. The annotation guidelines thus contain the ignorance taxonomy, along with all definitions and lexical cues for each taxonomy category, and include both positive and negative examples of statements of ignorance. The annotators reference these examples throughout the annotation task and they will be referenced here to illustrate our methods.

Both these positive and negative examples along with modified guidelines from BioScope (Vincze *et al.*, 2008) also help annotators identify the sentences that are statements of ignorance, also known as the *subject* of the lexical cues: the biomedical knowledge the lexical cue is qualifying. Our annotations are different from BioScope, but the scoping principles are similar: to capture the sentence fragment that is the subject of the task at hand. We chose to capture the full sentence that contains a lexical cue as the subject due to difficulties in capturing just fragments. Identifying these subjects will then allow for future work to identify the specific biomedical concepts that these statements of ignorance include using existing automatic concept recognition tools (e.g. Boguslav *et al.*, 2021). In the example above, the brackets < > signify the subject of the statement of ignorance, i.e. the full sentence. Note that there are no brackets around the negative example as it is not a statement of ignorance. The annotation guidelines describe how to remove or delete the annotation for the incorrect pre-marked cues and how to add the subject for the true statements of ignorance. For the full guidelines see: https://github.com/UCDenver-ccp/Ignorance-Question-Work.

### 2.5 Annotation task

For the annotation task itself, two independent annotators, Mayla R. Boguslav (M.R.B.) and Elizabeth K. White (E.K.W.) annotated 4–7 documents at a time checking in each article: the pre-marked cues, either deleting or adding the subject for those cues, and adding in any missed cues from the pre-processing. For example, using the positive example above, if the sentence ‘<however, there is CONTRADICTORY evidence from recent studies regarding the influence of IL-6 on insulin action and glucose metabolism> ’ was in an article, the cue CONTRADICTORY would be marked already based on the pre-processing and the annotator would note that, check that it should be mapped to *alternative options/controversy*, and add the subject as seen with the brackets here. On the other hand, if the negative example above, ‘although yin and yang are CONTRADICTORY in nature, they depend on each other for existence’, was in an article, CONTRADICTORY would be automatically marked through the pre-processing, however, the annotators would delete it as it does not signify ignorance. In terms of adding a missing cue, the phrase RECENT STUDIES from the positive example is not marked and signifies an *incomplete evidence*, where more evidence is needed. The annotators would recognize that and mark RECENT STUDIES to the narrow category of *incomplete evidence*, thus adding the cue to the taxonomy for the next round of annotation. Note that the subject of RECENT STUDIES would be the same as CONTRADICTORY in that sentence and would only be captured once for both cues, i.e. the full sentence.

To then determine if the annotations are reliable and create final gold standard documents, we evaluated the quality and agreement of the annotations per batch using inter-annotator agreement (IAA) measures (Hripcsak and Rothschild, 2005). The IAA is a measure of how well the annotations agree. Note that IAA does not bound the performance of classification algorithms, but is usually close to the limits (Boguslav and Cohen, 2017). Here, we calculated the F1 score between the two annotations, taking one annotation set as the ‘reference’ and calculating precision and recall with the other one (note that changing the ‘reference’ flips precision and recall, and thus F1 score remains the same). The F1 score then is the harmonic mean between precision and recall. The IAA was calculated on the exact text span of lexical cues or subjects chosen, as well as ignorance category assignments. We also calculate fuzzy IAA when the category assignments match but not the text span of the cue or the subject, or vice versa.

Spans of the lexical cues can overlap when annotators recognized differing numbers of words for multi-word cues. For example, the annotators might agree on the ignorance category but highlight either NEED or NEED TO BE in the sentence ‘Thus doses of D vitamin and calcium supplementation, which may differ from those recommended in normal pregnancy, NEED TO BE carefully tailored in thyroidectomised patients’. We take the maximum text span as the final span between the two annotators (NEED TO BE). This can also occur with the subject annotations, where the use of the Knowtator software may result in different text spans marked.

Category assignments can overlap when one person annotated to the ignorance category implied by the specific lexical cue while the other annotated to the lexical cue. In the end, we choose the narrowest applicable category or lexical cue in the taxonomy, to ensure we are capturing the correct information. For example, if one annotator mapped NEED TO BE in the above example to *future work* and the other mapped it to the lexical cue NEED TO BE which by subsumption implies *future work*, the final annotation would be the lexical cue. All of these fuzzy matches are resolved during the adjudication process to ensure a high-quality corpus. Ideally, IAA should be over 80% to trust the annotations and the reliability of the guidelines (Pustejovsky and Stubbs, 2012).

The final product of the annotation task is the gold standard corpus. To finalize and maintain the quality of the annotations, all disagreements were adjudicated, adjusting the guidelines and taxonomy accordingly with any newly identified cues along with the sentence or subject they were found in as a positive example. These discussions led to updates in all or some of the article annotations, the annotation guidelines, the ignorance taxonomy and the lexical cue list. This process was repeated each time incorporating new updates.

### 2.6 Automatic classification

**Fig. 2.**
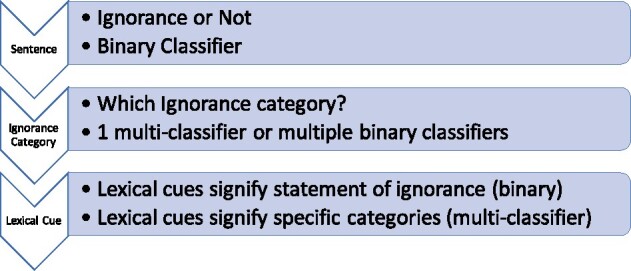
Classification flowchart. A flowchart of the different classification problems.

To determine if our task is feasible to automate, we test standard classifiers, with default settings, using the manually labelled dataset to evaluate performance (see Fig. 2). Any labelled sentences (subjects) or words (lexical cues) in the corpus are the positive samples to classify and any non-labelled sentences or words are negative samples. Our purpose is to demonstrate feasibility, not develop the optimal model, so a larger investigation of algorithms and tuned parameters will be the subject of future work with a larger corpus. As per the task description, classification can be made at the sentence or word level both as binary and multi-classification problems. At the sentence level, the binary task is to determine whether or not a sentence is a statement of ignorance, labelled in the corpus as subject. Then since each statement of ignorance has at least one lexical cue labelled, the sentence can also be labelled by the ignorance categories of its lexical cues. For example, the positive example above with lexical cues CONTRADICTORY and RECENT STUDIES (added as a new cue), would map to both *alternative options/controversy* and *incomplete evidence*. This now creates a multi-classification problem: to map the sentences to the specific ignorance categories of their lexical cues. Similarly, we can focus on the lexical cues, with the binary task to classify whether a word in an article is in a lexical cue or not as labelled in the corpus (e.g. the words CONTRADICTORY, RECENT, and STUDIES would be labelled as lexical cues). For the multi-classification task, the words would be mapped to the specific ignorance category [e.g. CONTRADICTORY to *alternative options/controversy* and (RECENT, STUDIES) to *incomplete evidence*]. For all classification tasks, we used a 90:10 split for the training and testing data and evaluated the models using F1 score.

For the binary sentence classification, we initially compared three different algorithms: logistic regression (LR), Gaussian Naive Bayes (GNB) and artificial neural networks (ANN) (Chollet *et al.*, 2015; Pedregosa *et al.*, 2011). Results from LR and GNB models were highly comparable to using ANN (data not shown) which we adopt here, due to the flexibility of ANN to build non-linear decision models (Dreiseitl and Ohno-Machado, 2002). Full-text documents from PMCOA were first split into sentences, which were then processed by the CountVectorizer package in python to tokenize words, remove punctuation and special characters, and produce a count vector of words as input to the ANN. The final tuned ANN had only two dense layers with dimensions 50 (to be proportionate with the large input shape) and 1. Overfitting was avoided by adding the training truncating functions (epochs ranged between 6 and 12). The batch size of 16 chosen for the training was small to allow for faster training and better generalization (Chien, 1994). We also applied the stratify function to make sure that the data splitting for the test is equal. Due to an imbalance of our data, we balanced it to the class with fewer samples before training to achieve a stable learning process and verified this decision by training on the non-balanced data in a separate trial.

The sentence multi-classification problem, classifying each ignorance sentence into a taxonomy category based on its lexical cues, has a similar setup to the sentence-binary task, using a vector of word counts as inputs. This task though is more complex because some sentences map to multiple categories, the categories are not balanced, and multi-classification problems are inherently harder than binary tasks. To combat all of these challenges, we decided to create a binary classifier for each taxonomy category that classifies all sentences as either the category of interest or not (meaning the other taxonomy categories). All classifiers, one for each taxonomy category, are run over all the data, allowing for one sentence to be classified in multiple categories easily (e.g. the positive example before would be easily classified as both *alternative options/controversy* and *incomplete evidence*).

The word-level classification tasks are to determine which words in a sentence indicate a lexical cue (binary task), and specifically for which taxonomy category (multi-classification). As our taxonomy is very similar to an ontology, we can use prior methods for concept recognition (Boguslav *et al.*, 2021) that explores different algorithms over many different ontologies. In particular, we make use of the best-performing span detection algorithms, namely CRF (Lafferty *et al.*, 2001) and BioBERT (Lee *et al.*, 2020), to determine the words in all lexical cues given an article. The CRF and BioBERT are trained for each specific iteration of the word-level classification. Thus, we first word-tokenize the articles using WordPunctTokenize in Python. We then assign each word a BIO- tag based on whether the word is at the beginning of a lexical cue (B), inside of it if it is a multi-word cue (I), outside of it meaning not a word in a lexical cue (O), or if the lexical cue contains a discontinuity (e.g. *NO...EXIST* where the ‘…’ signifies a discontinuity), we label the words in the ‘...’, that exist between the words of the lexical cue (O-). The input to the CRF and BioBERT to train then are the words with their target BIO- tag labels. When predicting, the input is the word-tokenized articles and the output is a BIO- tag for each word. To then determine what the lexical cue is, we re-assemble the BIO- tags by finding the B tags (single word lexical cues), combining the words with B and I in a row (multi-word lexical cues), ignoring the O labels (not lexical cues), and by combining B, I, and O- (discontinuous lexical cues). A more thorough discussion of BIO- tags can be found in Boguslav *et al.* (2021).

For the word-level binary task, we take every lexical cue from all ignorance categories, classify the words into BIO- tags, and then re-assemble them into the lexical cues. At the word level, the binary classifier can find new lexical cues that are not already in our lexical cue list by finding new combinations of words for example. So, we can compare the predictions of the classifier to our set of lexical cues in the taxonomy to determine if the classifier is finding new ones.

For the word-level multi-classification task, we explore two different methods. First, we created a model (CRF and BioBERT) for each ignorance taxonomy category following the previous work (Boguslav *et al.*, 2021). At the same time, however, BIO- tags have the capability to encode which category the word is from (i.e. B-*taxonomy category* or I-*taxonomy category*), and so we can create one multi-classifier (CRF or BioBERT) for the word-level classification as well.

## 3. Results

### 3.1 Statements of ignorance employ a rich vocabulary

Manual review of 736 paper abstracts to develop a taxonomy of ignorance based on implied knowledge goals revealed that abstracts contained on average seven (minimum 0, maximum 24) statements of ignorance, involving 897 lexical cues. Subsequent refinement of 60 full-text articles during the annotation task and hedging cues from previous work added many more lexical cues totalling to 1 890 in the end. Organizing the lexical cues by their knowledge goals led to the final taxonomy with five broad categories in italics, composed of 13 narrow ones:

- *Question Answered by this Work*
- *Levels of Evidence*: full unknown, explicit question, incomplete evidence, superficial relationship, probable understanding
- *Anomaly/Curious Finding*
- *Barriers*: alternative options/controversy, difficult task, problem/complication
- *Future Opportunities*: future work, future prediction, important consideration

Note that both *question answered by this work* and *anomaly/curious finding* are considered as categories at both the broad and narrow levels. As for the three broad categories that unify multiple narrow categories, ‘Levels of Evidence’ contains statements that answer the questions of how much evidence we have and how confident we are in that evidence, in increasing order. ‘Barriers’ contains statements of obstacles, complications, or multiple options that prevents research from moving forward and needs to be overcome. ‘Future Opportunities’ includes statements of future needs such as future work or considerations. These broad categories help simplify the 13 categories for annotation purposes.

These 13 narrow categories, after the full annotation task, contain 1 890 lexical cues that can signify a statement of ignorance (Table 1). This signifies that the language used to articulate similar knowledge goals is rich and varied. *Incomplete evidence* contains the most cues. Interestingly, most ignorance statements are not presented as an explicit question with a question mark or a question word (who, what, where, etc.), as *explicit question* has the fewest number of cues compared to all other categories. Yet these *implicit* questions still imply a question and still present a knowledge goal. The remaining 12 ignorance categories are defined by their knowledge goals, from finding more evidence for *incomplete evidence* to creating new methods for a *difficult task*.

**Table 1.** The ignorance taxonomy with definitions, knowledge goals, example cues, and total cue count.

Ignorance category	Definition	Knowledge goal	Example cues	Total cues
**Question answered by this work**	A statement of a goal or objective of a study that is attempted or completed during the study.	To find the answer(s) in the article; determine if the question(s) is (are) fully answered in the article	Aim, goal, objective, our study, sought, to determine	58
Full unknown	A statement that indicates something is not known (a lack of information), or information is presented for the first time (new or novel) and a significant amount of research is needed; not a statement about the absence of something.	To explore the unknown further to gain any insights	Could not find, do not know, elusive, not…established, uncertain, still unclear	137
Explicit question	An explicit statement of inquiry (with a question mark or question word such as how, where, what, why).	To find answers to the question and/or discover methodologies that will help answer the question	?, what, where, wondered, why	17
Incomplete evidence	A positive or negative statement proposing a possible/feasible explanation for a phenomenon on the basis of limited evidence as a starting point for further investigation OR a statement that information is needed to support an assertion or claim, including both positive and negative statements. Either a statement that some evidence already exists, explaining how current findings support previous work, adding confidence to a claim OR a statement that information is limited, more research is needed or is ongoing including limitations—biases or short comings related to the study design and execution.	To gather more evidence to support the claim OR conduct more research to determine the validity of the claim; complete the partial picture; consider the short comings and biases for the next experiment and how it can be addressed.	A good understanding, believe, evidence…limited, has been suggested, hypothesis, no studies, possibly, preliminary stage, remains under investigation, still being discovered, support, trend	619
Superficial relationship	A statement about a connection, link or association between at least two variables; connectedness between entities and/or interactions representing their relatedness or influence.	To confirm the connection, link or association between variables; determine the full underlying relationship between variables	Affect, associated, correlate, factor, influence, interact, link, pattern, tend	133
Probable understanding	A statement staking a claim to the most likely explanation, relationship, or phenomenon; assumes that there is a good chance this understanding is correct.	To determine if the most likely option is correct or if another option is more feasible	Almost all, assumed, concluding, evident, it is clear, most likely, thus	119
**Anomaly/curious finding**	A statement of a surprising result, conclusion, observation or situation; the researchers were not expecting the result, conclusion, observation or situation but are intrigued by it.	To explore the surprising result, conclusion or situation more and determine if the result, conclusion, observation or situation is repeatable	Appeared to be, interestingly, noteworthy, surprisingly	89
Alternative options/controversy	Either an explicit statement of multiple (at least 2) choices, actions, approaches or methods that need to be experimentally determined, including statements with an implied second option, such as ‘whether’. This includes a statement of disagreement amongst researchers OR a lack of consensus OR at least two possible answers presented as results from different researchers—usually in reference to previous results and stated when results disagree with each other OR contradictions.	To determine the correct option or a better option and if there are disagreements, or to determine the truth to break any disagreements	Cannot rule out, claims, has been challenged, whether, whilst	193
Difficult task	A statement of something not easily done, accomplished, comprehended or solved; or a complicated thing with a multitude of underlying pieces or parts; heterogeneity; excludes medical complications.	To create methods to study the complicated system and to better understand any piece of the complicated system; potentially requires new experiments or better techniques	Not feasible, remains…challenge, variability, rarely able to	69
Problem/complication	A statement of issues, problems, mistakes or medical complications that are cause for anxiety and/or worry.	To determine the gravity of the concern and determine if it needs to be dealt with before the next experiment or study	Issue, error, insufficient, lack of reproducibility, publication bias, underestimated	86
Future work	A statement of extensions, including next steps, directions, opportunities, approaches or considerations of the described work that may be implemented at some future time point. This also includes a statement of suggestion or a proposal as to the next best course of action, especially one put forward by an authoritative body; advice telling someone the best action to take.	To determine the next course of action based on this future work proposal	Additional research, are needed, continue to explore, further study, more…studies, recommend, warrants, worthy of closer attention	201
Future prediction	A statement of extrapolation of given data into the future and/or from past observations, without reference to next steps.	To run the simulation or experiment to determine if the prediction is correct; publicize the outcomes of the study to the correct people	Allow, expect, if so, serve as a basis, will	17
Important consideration	A statement calling for attention including an action needed to be taken immediately or information that needs to be disseminated immediately OR critical: being in or verging on a state of crisis or emergency OR urgently needed OR absolutely necessary.	To take the urgent action ASAP or distribute the knowledge ASAP	Call for action, cautious, crucial, emphasis, global problem, high on the agenda, necessary, relevant to note, vital	152

*Note:* The categories in bold are both broad and narrow categories.

### 3.2 Robust annotation guidelines yield a high-quality corpus

Annotators were asked to identify statements of ignorance in 60 full-text articles about prenatal nutrition from PMCOA in batches of 4–7 articles for a total of 13 batches. The annotation guidelines described above were provided that specified the ignorance taxonomy, all definitions and lexical cues for each taxonomy category, and included positive and negative examples of statements of ignorance for each category.

The positive and negative examples for each lexical cue were very helpful in making the annotation task more feasible. Surprisingly, almost every cue contained a negative example. Even for seemingly obvious cues such as UNKNOWN, there is a negative example. A statement such as ‘to make inference, the maximum likelihood method is applied to estimate the UNKNOWN parameters in the empirical log-odds ratio models given in (3)-(6)’ is a negative example in that it is a description of a methodology that helps determine missing parameters. Also, many new lexical cues were added during the annotation task, and to reflect that the guidelines were updated.

After each of the 13 batches, IAA was assessed for concordance in span indicated for the lexical cue or subject and for category assignment, calculating both exact match IAA and fuzzy match IAA for each. Overall, we present a high-quality corpus with robust annotation guidelines. Combining all articles, we can reliably annotate statements of ignorance: we achieve a category IAA of 78%, subject IAA of 87%, fuzzy category IAA of 79% and fuzzy subject IAA of 90%. Thus we achieved an IAA around 80% for the ignorance category and around 90% for the subject.

### 3.3 The scientific literature is rich in statements of ignorance

The scientific literature, exemplified by prenatal nutrition, is rife with statements of ignorance (see Table 2). There are over 10 000 lexical cue annotations included in nearly 4 000 subject annotations in 60 articles. These articles include 7 304 sentences and 249 133 words. The majority of these category annotations refer to *incomplete evidence* or *superficial relationship*. For each ignorance category, the average number of annotations per article ranges from 1 to 60, with the median from 1 to 40. Also, not every article includes every ignorance category (the minimum number of annotations per article is zero). However, there are articles with over 100 annotations to one category. Thus, each article contains many statements of ignorance that map to all the taxonomy categories.

**Table 2.** Per article counts of annotations

Category	# Total annotations	Average # annotations	Median # annotations	Maximum # annotations
Question answered by this work	310	5.17	2	23
Full unknown	191	3.18	1	20
Explicit question	84	1.4	0.5	27
Incomplete evidence	3 628	60.47	39.5	330
Superficial relationship	1 953	32.55	18	161
Probable understanding	749	12.48	4.5	107
Anomaly/curious finding	501	8.35	4	39
Alternative options/controversy	933	15.55	7.5	94
Difficult task	164	2.73	1	25
Problem/complication	352	5.87	2.5	30
Future work	535	8.92	3.5	79
Future prediction	173	2.88	1	29
Important consideration	717	11.95	4	119
All categories	10 289	171.5	126.5	1 021
Subject	3 852	64.2	56.5	262

*Notes:* Total number of annotations in all articles and statistics per article. Note that all categories except for ALL CATEGORIES and SUBJECT (have 1) have zero minimum number of annotations.

Even with so many lexical cue annotations, many of them are repeats of the same cue (see Table 3). Only 8% of the 10 000 annotations are unique lexical cues. Furthermore, only 43% of all cues collected (1 890) are used in the articles, suggesting that there are many ways to say knowledge is missing or incomplete, with many specific lexical cues used more often than others. Still, the trends of the unique counts are the same as the full counts of annotations. *Incomplete evidence* and *superficial relationship* still comprise the majority of the annotations.

**Table 3.** Per article unique counts of annotations

Category	# Total unique annotations	Average # unique annotations	Median # unique annotations	Maximum # unique annotations
Question answered by this work	36	3.13	2	10
Full unknown	47	2.1	1	7
Explicit question	12	0.87	0.5	5
Incomplete evidence	268	25.28	22	84
Superficial relationship	84	10.88	9.5	45
Probable understanding	51	4.47	3	22
Anomaly/curious finding	43	3.85	2	14
Alternative options/controversy	77	6.17	4.5	24
Difficult task	28	1.85	1	11
Problem/complication	32	2.78	1	13
Future work	67	4.52	3	20
Future prediction	11	1.47	1	6
Important consideration	50	4.12	3	26
All categories	806	71.5	66.5	273

*Notes:* Total number of unique annotations in all articles and statistics per article. Note that all categories except for ALL CATEGORIES (has 1) have zero minimum number of unique annotations.

The frequency of lexical cue annotations varies based on the article section (see Table 4). As might be intuitive, the conclusion contains the most annotations, followed by the discussion, and then the method section. The abstract section contains the fewest annotations, which may be due to the normally small size of this section compared to the others. On average, each section has at least 2 annotations and at most 117 annotations. The medians are slightly lower, indicating some outlier articles with many annotations. In fact, the maximum number of annotations in one section in one article is 524 in the conclusion section. Aside from the method and conclusion sections, there exist articles with annotations in some sections but not all. For each article with a conclusion section, there are at least four annotations. Similarly, every article with a method section has an annotation in that section. For example, ‘OWING TO the LACK OF other available DATA, we used the annual number of live births in Iaşi county REPORTED on 01 July 2009 (*n* = 9499) to define the size of the reference group [23]’ with OWING TO mapping to *problem/complication*, LACK OF…DATA mapping to *full unknown*, and REPORTED mapping to *incomplete evidence*. The sentence presents a method while also explaining why that particular one due to a lack of data. Thus, looking within the article, the different sections contain differing numbers of statements of ignorance.

**Table 4.** Per article annotation counts per section

Section	# Total articles	# Total annotations	Average # annotations	Median # annotations	Minimum # annotations	Maximum # annotations
Abstract	42	80	1.9	1	0	29
Introduction	55	1416	25.75	14	0	571
Methods	35	1403	40.09	30	1	367
Results	29	323	11.14	6	0	46
Discussion	31	1940	62.58	37	3	258
Conclusion	28	5127	183.11	107.5	4	990

*Notes:* Total number of annotations by section in all articles with section delineation and statistics per article.

### 3.4 Statements of ignorance and lexical cues can be automatically identified

We can automatically identify statements of ignorance (see Table 5) and lexical cues (see Table 6). Due to limited space, however, we only show the top algorithms for classification with both the training and testing F1 scores. For binary sentence classification (is a sentence labelled as subject or statement of ignorance in the corpus or not), we achieved an F1 score of 0.85 with 7 epochs for the ANN.

**Table 5.** Sentence classification both binary and all 13 categories

Ignorance category	Training F1 score	Training support	Testing F1 score	Testing support
All categories binary	0.97	3 390	0.85	377
Question answered by this work	>0.99	1 965	0.89	109
Full unknown	>0.99	2 223	0.90	123
Explicit question	0.99	2 782	0.84	155
Incomplete evidence	>0.99	1 389	0.90	77
Superficial relationship	>0.99	3 288	0.87	183
Probable understanding	>0.99	2 179	0.86	121
Anomaly/curious finding	>0.99	1 288	0.87	72
Alternative options/controversy	>0.99	1 416	0.89	79
Difficult task	>0.99	532	0.89	30
Problem/complication	>0.99	988	0.81	55
Future work	>0.99	1 270	0.88	71
Future prediction	>0.99	489	0.94	27
Important consideration	>0.99	1 677	0.82	93

*Notes:* Note that one sentence can map to more than one category and so they will not add up to the total binary.

**Table 6.** Word classification both altogether, binary, and to all 13 categories

Ignorance category	Training F1 score	Training support	Testing F1 score	Testing support
All categories binary	0.95	11 552	0.93	1 210
Question answered by this work	0.91	474	0.83	51
Full unknown	0.85	299	0.8	28
Explicit question	0.98	68	0.9	15
Incomplete evidence	0.96	4 342	0.96	520
Superficial relationship	0.98	1 812	0.99	199
Probable understanding	0.96	753	0.95	73
Anomaly/curious finding	0.91	522	0.94	61
Alternative options/controversy	0.92	961	0.9	117
Difficult task	0.87	199	>0.99	19
Problem/complication	0.93	415	0.94	36
Future work	0.91	716	0.87	84
Future prediction	0.97	180	0.94	18
Important consideration	0.98	737	0.97	85
All categories combined	0.84	9 416	0.85	1 073

For the sentence multi-classification problem, we created 13 binary models with the target class against the other 12 classes (e.g. *difficult task* class versus the rest of the classes). As each category had fewer positive examples than the other categories combined, we balanced the data and noticed that in the non-balanced data the F1 score is biased towards the class that has the higher number of samples (data not shown). For the balanced trials, testing F1 scores ranged between 0.8 and 0.94 (see Table 5).

Going deeper to identify the specific lexical cues using BIO- tags on the word level, BioBERT performed better than the CRF for all word-level classification tasks (see Table 6 reporting only BioBERT). The binary task (ALL CATEGORIES BINARY) achieved an F1 score of 0.93 and contains the most data of all the tasks. For the 13 binary classifiers created for the multi-classification task to each narrow category, the F1 scores ranged between 0.8 to greater than 0.99 based on varying amounts of data per category. Combining all these data into one multi-classifier using the BIO- tag capability to add a category (e.g. B-*incomplete_evidence*), we achieved an F1 score of 0.85. More data are necessary to truly validate these results but they do seem promising to show that we can automatically identify statements of ignorance.

For all tasks, F1 scores are above 0.8 and rise even higher for categories with more data. Furthermore, both the combined and binary methods performed quite well. For normalizing the binary method over all the data including training and testing, all lexical cues predicted mapped to the taxonomy except for about 6%. As the dictionary of cues map each cue to only one ignorance category, this number is quite low and contributes to an F1 score of 0.90 to all categories. The errors seemed to be due to minor variations on the ignorance cues already in the taxonomy, such as plurals or words in different orders. More data are necessary to truly validate these results, but they do seem promising.

## 4. Discussion

Identifying and categorizing statements of ignorance in terms of their knowledge goal is an important new NLP task that formalizes and extends prior work in uncertainty, hedging, speculation, and meta-knowledge. Our ignorance taxonomy subsumes prior work by Firestein (2012), who elucidates a few types of ignorance informally including curiosity, possibility and controversy, which extend to *anomaly/curious finding*, *incomplete evidence*, and *alternative options/controversy* in our taxonomy, respectively. Kuhn (2012) discusses how small discrepancies in the predictions of a theory can accumulate until they cause a crisis necessitating a completely new theory. In our taxonomy, these correspond to the category of *anomaly/curious finding*. Pearl and Mackenzie (2018) aimed to forge connections from correlation to causation, the *superficial relationship* category in our taxonomy, since the literature is filled with associations. Han *et al.* (2011) focused on healthcare with categories including probability, ambiguity, and complexity extending to *probable understanding*, *anomaly/curious finding* and *difficult task* in our taxonomy, respectively. Smithson’s (2012) taxonomy includes incompleteness and probability, which map to *incomplete evidence* and *probable understanding* in our taxonomy, respectively. Kilicoglu *et al.* (2017) using SemRep include the categories probable and possible, which fall under *probable understanding* and *incomplete evidence* in our taxonomy, respectively. Many of these works also provide lexical cues to help do their tasks, which we extend into nearly 2 000 cues. To the best of our knowledge, this is the largest lexical cue list and shows that statements of ignorance employ a rich vocabulary.

Using our ignorance taxonomy and lexical cues, we provide robust annotation guidelines that yield a high-quality corpus. Human readers can reliably identify and categorize statements of ignorance as demonstrated in our annotation task with all IAAs over 80%. Furthermore, this corpus highlights how rich the scientific literature is with statements of ignorance (see Tables 2–4), both by article and by section. Also, most statements of ignorance are implicit (the *explicit question* category is quite small), which makes this task difficult. As in prior work (e.g. Farkas *et al.*, 2010; Vincze *et al.*, 2008), we provide a very detailed ignorance taxonomy, extensive annotation guidelines, and our gold standard corpus.

We also can automate this task by creating classifiers to identify statements of ignorance and lexical cues. Although we did not exhaustively train and tune our models, our results look promising that this task is feasible (see Tables 5 and 6). We achieved F1 scores above 0.8 for all classification tasks. Furthermore, in the binary word-level classification task to identify any lexical cue that signifies a statement of ignorance, we may be generating new lexical cues beyond our list, as 6% of the predicted cues did not match our list.

The major limitations of this work are the small amount of data and the focus on prenatal nutrition. The corpus contains 60 articles which is enough to train classification models, but not enough to conduct an external validation. Future work currently underway includes more annotations to create a separate evaluation set. It further remains to be seen if these results generalize beyond the field of prenatal nutrition. Even with these limitations though, our results are quite promising for both statements of ignorance (sentences) and the specific lexical cues in prenatal nutrition. It is both important to be able to identify statements of ignorance and lexical cues to formally represent and compute over these sentences along with other biomedical ontology terms such as genes, proteins and diseases. Furthermore, if a sentence maps to more than one ignorance category, identifying the specific lexical cues can distinguish between the categories. All training and testing F1 scores are above 0.8, which according to Pustejovsky and Stubbs (2012), is an ideal agreement level, showing that our data are reliable.

Even with these limitations, discussions of these statements of ignorance can both refine and improve research questions, identify established facts, and facilitate the comparison of approaches for further research. These discussions of scientific questions are of interest to researchers, educators, publishers, and funders because they provide insights and directions for new research and may provide context for existing results. Formalizing and disseminating such statements of ignorance in the scientific literature is an important new NLP task with the potential to greatly impact how we view the literature and scientific progress in general.

## 5. Conclusion

The new NLP task of finding scientific questions or statements of ignorance in the scientific literature will not only yield novel text-mining tools, but will also trace out the evolution of scientific thought in a discipline, point out gaps or flaws in existing theories, and provide new avenues for future insights. Here, we not only showed that this task is feasible but also created an ignorance taxonomy, a gold standard corpus, and promising preliminary classification models. The goal is to help enhance literature awareness by creating an *ignorance-base* (compared to a knowledge-base) of statements of ignorance found in the literature for all to explore, including students, researchers, publishers, and funders.

## Software and data availability

Corpus and source code are freely available for download at https://github.com/UCDenver-ccp/ Ignorance-Question-Work. The source code is implemented in Python.

## Author contributions

M.R.B., S.M.L. and L.E.H. conceived of the ideas, M.R.B. and E.K.W. annotated all documents for the corpus, M.R.B. and N.M.S. created the classification models, and all authors helped write and reviewed the manuscript.
